# MicroRNA-4443 Causes CD4+ T Cells Dysfunction by Targeting TNFR-Associated Factor 4 in Graves’ Disease

**DOI:** 10.3389/fimmu.2017.01440

**Published:** 2017-11-01

**Authors:** Yicheng Qi, Yulin Zhou, Xinxin Chen, Lei Ye, Qianwei Zhang, Fengjiao Huang, Bin Cui, Dongping Lin, Guang Ning, Weiqing Wang, Shu Wang

**Affiliations:** ^1^Shanghai Clinical Center for Endocrine and Metabolic Diseases, Department of Endocrinology and Metabolism, Ruijin Hospital, Affiliated to Shanghai Jiao-Tong University School of Medicine, Shanghai, China; ^2^Department of Endocrinology and Metabolism, Shanghai Ninth People’s Hospital, Affiliated Shanghai Jiao-Tong University School of Medicine, Shanghai, China

**Keywords:** Graves’ disease, CD4+ T cells, microRNA-4443, TNFR-associated factor 4, NF-κB

## Abstract

**Context:**

Aberrant CD4+ T cell function plays a critical role in the process of Graves’ disease (GD). MicroRNAs (miRNAs) are important regulators of T cell activation, proliferation, and cytokine production. However, the contribution of miRNAs to CD4+ T cell dysfunction in GD remains unclear.

**Objective:**

To investigate how certain miRNA causes aberrant CD4+ T cell function in GD patients.

**Methods:**

We compared the expression pattern of miRNAs in CD4+ T cells from untreated GD (UGD) patients with those from healthy controls. The most significantly dysregulated miRNAs were selected and their correlations with clinical parameters were analyzed. The effect of miR-4443 on CD4+ T cells cytokines production and proliferation was assessed. The potential gene target was identified and validated.

**Results:**

GD patients had unique pattern of miRNA expression profile in CD4+ T cells comparing to healthy subjects. miR-10a, miR-125b, and miR-4443 were the three most significantly dysregulated miRNAs. The elevated miR-4443 levels were strongly correlated with clinical parameters in an independent dataset of UGD patients (*N* = 40), while miR-4443 was normally expressed in GD patients with euthyroidism and negative TRAb level. We found that miR-4443 directly inhibited TNFR-associated factor (TRAF) 4 expression to increase CD4+ T cells cytokines secretion as well as proliferation through the NF-κB pathway. Furthermore, the TRAF4 levels in GD patients were inversely correlated with miR-4443, and knocking down TRAF4 had a similar effect with miR-4443 overexpression.

**Conclusion:**

The increased expression of miR-4443 induced CD4+ T cells dysfunction by targeting TRAF4, which may cause GD.

## Introduction

Graves’ disease (GD) is a common immune-mediated disease. Lymphocytic infiltrations in thyroid lead to the production of autoantibody against thyrotropin receptor [thyroid stimulating hormone (TSH) receptor antibody (TRAb)], which then mimics the action of TSH, causing excessive thyroid hormone production and hyperthyroidism (1).

The imbalance of Th1/Th2 cells and Treg/Th17 cells may alter the levels of pro- and anti-inflammatory cytokines, thus contributing to GD onset and development (2). Th1 cell could produce IFN-γ and enhance CXC chemokines production in GD (3). Th2 cell could produce IL-4, IL-5, IL-10, and costimulatory molecules, in turn increase B cell differentiation and antibodies release in GD (4). The proportion of Th17 cells increased in intractable GD patients (5). Our previous study found chemokine (C-C motif) ligand 20 (CCL20) was upregulated in CD4+ T cells from GD patients through NF-κΒ and MAPK pathways (6). CCL20 is a potent chemoattractant for Th17 cells and is closely related to IL17 signal activation. Additionally, we found CD40L overexpression on CD4+ T cells surface which serves as the downstream effector of osteopontin to produce immunoglobulin in GD (7). These findings suggested the dysfunction of CD4+ T cells played an important role in the pathogenesis of GD development. However, the potential mechanisms underlying CD4+ T cell dysfunction need to be clarified.

MicroRNAs (miRNAs) are important regulators of multiple immune pathways (8). Dysfunctions of miRNAs have been indicated in many autoimmune diseases. For example, decreased miRNA-142-3p/5p levels in CD4+ T cells caused T cell activation and B cell hyperstimulation in systemic lupus erythematosus (9). In rheumatoid arthritis, miR-146a was upregulated and T cell apoptosis was suppressed (10). In multiple sclerosis, miR-17 expression in CD4+ T cells was associated with natalizumab treatment and disease relapse (11). However, the miRNA expression profile in CD4+ T cells of GD patients and the pathogenesis underlying miRNA dysregulation needs further investigation.

In this work, we profiled the expression pattern of miRNAs of CD4+ T cells in GD patients and how they are related to GD development.

## Subjects and Methods

### Subjects

Forty untreated GD (uGD) patients, 30 euthyroid GD (eGD) patients, 18 TRAb negative-conversion GD (nGD) patients, and 30 age- and sex-matched healthy control donors (hCD) were enrolled from Ruijin Hospital affiliated to Shang-hai Jiao Tong University School of Medicine. All of the uGD patients, without previous treatment, were newly diagnosed through patients’ history, clinical manifestation, and laboratory examination. The presence of typical manifestation including heat intolerance, fatigue, increased appetite, increased sweating, weight loss, muscle weakness, tremors, and thyroid gland was diffusely enlarged. The abnormal result of laboratory examinations included high free T3 (FT3), FT4, and low sensitive TSH (sTSH) as well as high TRAb. eGD patients were treated with methimazole (MMI) for 2–4 months and reached normal FT3 and FT4 levels. nGD patients were treated with MMI until TSH, FT3, FT4, and TRAb levels returned to the normal range and remained stable for at least 3 months. Healthy subjects without any past or present history of thyroid disease were enrolled in this study. The subject characteristics and clinical information are shown in Table 1. The study was approved by the Research Ethics Board of Ruijin Hospital. Informed written consents were obtained from each participant.

**Table 1:** Clinical characteristics of the subjects.

	hCD	uGD	eGD	nGD
*n* (male/female)	30 (10/20)	40 (14/26)	30 (8/22)	18 (4/18)
FT3 (pmol/l)	4.35 ± 0.50	27.95 ± 13.67^a^	4.45 ± 0.77^b^	4.17 ± 0.42^c^
FT4 (pmol/l)	13.34 ± 1.33	41.06 ± 12.83^a^	11.67 ± 2.90^b^	12.98 ± 2.20^c^
sTSH (mIU/l)	1.93 ± 0.79	0.003 ± 0.006^a^	2.10 ± 3.51^b^	1.90 ± 0.83^c^
TRAb (IU/l)	0.38 ± 0.25	17.33 ± 12.32^a^	9.22 ± 8.30	0.72 ± 0.35^c^
TPOAb (IU/ml)	0.28 ± 0.24	328.54 ± 317.2^a^	358.97 ± 301.53	55.59 ± 65.66^d^
TGAb (IU/ml)	1.03 ± 0.64	273.15 ± 345.57^a^	139.18 ± 209.32	73.90 ± 65.66^d^

*^a^*P* < 0.01, untreated GD (uGD) compared with healthy control donors (hCD)*.

*^b^*P* < 0.01, euthyroid GD (eGD) compared with uGD*.

*^c^*P* < 0.01, TRAb negative-conversion GD (nGD) compared with uGD*.

*^d^P < 0.05, nGD compared with uGD*.

### CD4+ T Cell Isolation

Fresh peripheral blood mononuclear cells (PBMCs) from GD patients and healthy donors were isolated by Ficoll-Paque centrifugation (Sigma Aldrich, St. Louis, MO, USA). For the purification of CD4+ T cells from fresh PBMCs, positive selection by human CD4 Micro Beads (Miltenyi Biotec, Bergisch Gladbach, Germany) was used according to the manufacturer’s instructions. The purity of CD4+ T cells was >95% as analyzed by flow cytometer (BD Biosciences, Bedford, MA, USA). The isolated CD4+ T cells were used for further research [microarray analysis, real-time reverse transcription–polymerase chain reaction (qRT-PCR), and culture].

### miRNA Microarrays

CD4+ T cell samples from three uGD patients and three hCD were used for microarray analysis, and the miRNA profiles were compared between the GD and control group. miRNA microarray profiling was performed using Agilent Human miRNA (8*60K) V19.0 (Santa Clara, CA, USA) according to the manufacturer’s recommendations. Briefly, miRNA molecular in total RNA was labeled using the miRNA Complete Labeling and Hyb Kit. After labeling, each slide was hybridized with 100 ng Cy3-labeled RNA using miRNA Complete Labeling and Hyb Kit. Next, the slides were washed in staining dishes with Gene Expression Wash Buffer Kit. The slides were then scanned using the Agilent Microarray Scanner and Feature Extraction software 10.7 with default settings. Raw data were normalized by the Quantile algorithm in the Gene Spring Software 11.0.

### T Cell Culture and Transfection

CD4+ T cells were rested in RPMI 1640 medium (Gibco, Carlsbad, CA, USA) plus 10% FBS and 1% penicillin/streptomycin overnight. For transfection, CD4+ T cells were transfected with 100 nM miRNA mimics or negative controls (Genepharma, China), 300 nM miRNA inhibitors or inhibitor negative controls (Genepharma), and 300 nM small-interfering RNAs targeting human TNFR-associated factor (TRAF) 4 or negative controls (Ribobio, China) using Lipofectamine 3000 (Invitrogen, Grand Island, NY, USA), according to the manufacturer’s protocol. To evaluate the transfection efficiency, these oligonucleotides were labeled with Cy3. Six hours after transfection, we evaluated the transfection efficiency by fluorescence microscope. The percentages of Cy3 positive cells were about 60%. Twelve hours after transfection, the cells were stimulated with 5.0 µg/ml anti-CD3 and 5.0 µg/ml anti-CD28 mAbs (eBioscience, San Diego, CA, USA); the cells and supernatants were then collected for further analysis 48 h later.

### Real-time Reverse Transcription–Polymerase Chain Reaction

Total RNA was isolated using Trizol reagent (Invitrogen). cDNAs were synthesized from whole cellular RNA using a miScript Reverse Transcription Kit (Qiagen, Hilden, Germany). The expression levels of miRNAs were confirmed with a miScript SYBR Green PCR kit and miRNA-specific primers (Qiagen). RNU6-2 was used as the normalization control, and the Light Cycler 480 software (Roche Applied Science, Indianapolis, IN, USA) was used to analyze data. The quantity of mRNA was determined by reverse transcription using PrimeScript RT reagent Kit (Takara, Shiga, Japan) and real-time PCR with SYBR Premix Ex Taq (Takara) and normalized to β-actin. The gene specificity of all of the primers was confirmed by BLAST searches. The primers are presented in Table S1 in Supplementary Material. All of the reactions were performed in triplicate and the results were calculated by the ΔΔCt value and normalized against endogenous controls.

### ELISA

The concentrations of the indicated cytokines were measured quantitatively using ELISA kits according to the manufacturer’s procedure. The ELISA kits were purchased from R&D Systems (Minneapolis, MN, USA). The optical density values were read at 450 nm.

### Western Blot Analysis

Cell lysates were subjected to western blot analysis according to standard protocols. After blocking, the membranes were incubated overnight at 4°C with primary antibodies to TRAF4 (Abcam, Cambridge, UK), p65, phosphorylated p65, IκBα, or phosphorylated IκBα (Cell Signaling Technology, Danvers, MA, USA). GAPDH (Cell Signaling Technology) was used as a normalized control. Next, the membranes were incubated with horseradish peroxidase-conjugated secondary antibody (Cell Signaling Technology). Protein bands were illuminated using ECL Prime Western Blotting Detection Reagent (GE Healthcare, Little Chalfont, Buckinghamshire, UK).

### Cell Proliferation Assays

After transfection with the mimics, inhibitors or controls for 12 h, human peripheral blood CD4+ T cells were plated in a 96-well plate and were activated with anti-CD3 and anti-CD28 mAbs. At the appropriate time, the cells were incubated with 10 µL of CCK-8 (Dojindo, Kumamoto, Japan) per well for 3 h at 37°C. The absorbance was determined at 450 nm using an enzyme-labeled instrument (Thermo Scientific, Rockford, IL, USA).

### Luciferase Activity Assay

The psiCHECK-2 vector (Promega, Madison, WI, USA) was used to clone the TRAF4 3′UTR sequence containing the putative miR-4443 binding sites, designated as wild type (WT). Because two putative miR-4443 binding sites were predicted in the TRAF4 gene, reporter plasmids of the corresponding mutation (Mut1 and Mut2) and both sites mutation (Mut3) were constructed. HEK293T cells were seeded in 24-well plates the day before transfection. For each well, 100 ng of wild-type or mutant TRAF4 3′-UTR psiCHECK-2 plasmid was transiently cotransfected with miRNA mimics or negative controls using Lipofectamine 2000 (Invitrogen). Cell lysates were harvested 48 h after transfection, and the cells were subjected to a Dual-Luciferase Reporter Assay System (Promega) according to the manufacturer’s instructions. Renilla luciferase activities were normalized to firefly luciferase activities to control for the transfection efficiency.

### Statistical Analysis

SPSS version 17.0 and Graph Pad Prism 5.0 were applied in the statistical calculations. Student’s *t*-test or the Mann–Whitney *U*-test was performed to compare the differences between the two groups. Correlations between the different variables were analyzed by simple correlation using Spearman’s test. Multivariate analysis was performed by multiple linear regression analysis using miR-4443 as a dependent variable, and variables that were significant at *P* < 0.20 level in Spearman’s correlations were used as covariates. Data are presented as mean ± SD. *P* < 0.05 was considered significant.

## Results

### Upregulated miR-4443 Expression in CD4+ T Cells of uGD Patients

**Figure 1:**
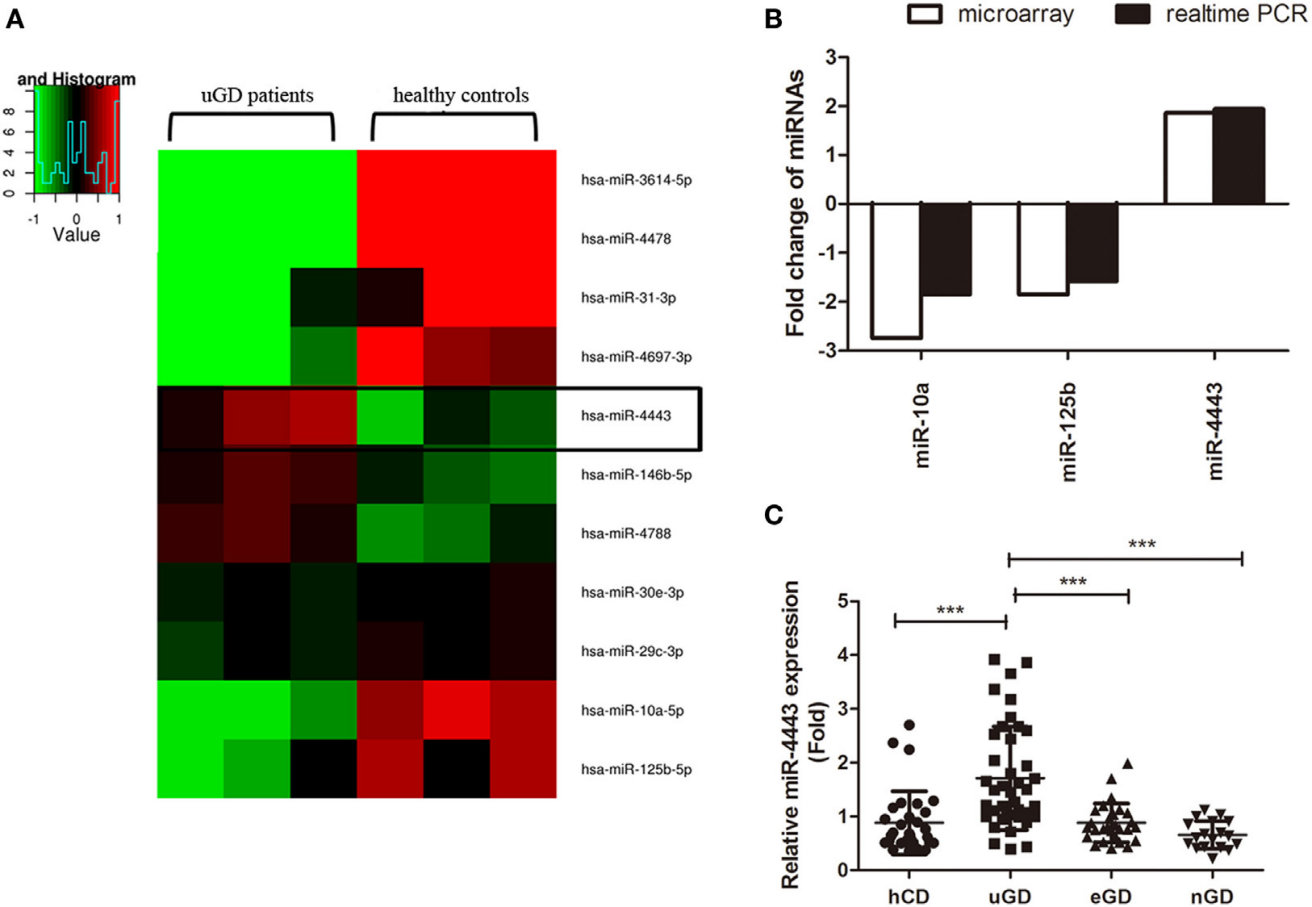
Increased expression of microRNA (miR)-4443 in CD4+ T cells from untreated Graves’ disease (uGD) patients. **(A)** Heat map of differentially expressed miRNAs (*P* < 0.05) in CD4+ T cells from three uGD patients and three healthy control donor (hCD). **(B)** Differential miR-10a, miR-125b, and miR-44443 expression in CD4+ T cells was verified by real-time polymerase chain reaction (PCR) using a larger GD and control sample. **(C)** Expression of miR-4443 in CD4+ T cells from healthy controls, uGD patients, euthyroid GD (eGD) patients and TRAb negative-conversion GD (nGD) patients. Bars show the mean ± SD. **P < 0.01; ***P < 0.001.

We assessed 2006 human miRNAs in CD4+ T cells from three uGD patients and three healthy controls with Agilent Human miRNA array. As shown in Figure 1A, we found 11 miRNAs displayed significant differences (*P* < 0.05). Among them, eight miRNAs were suppressed, whereas three miRNA were enhanced in GD CD4+ T cells compared with normal CD4+ T cells. Based on fold change greater than 1.5 and the relative expression level, miR-10a, miR-125b, and miR-4443 were selected for real-time PCR validation. Consistently, we found that miR-10a (*P* < 0.001) and miR-125b level (*P* = 0.0014) was decreased and miR-4443 level (*P* < 0.001) was increased in an independent dataset (40 uGD and 30 controls, Figure 1B). Interestingly, the miR-4443 level was strongly correlated with FT3, FT4, and independently with TRAb (Table 2). In euthyroid group and TRAb negative-conversion group, the miR-4443 level was reduced to normal levels (eGD vs. hCD, *P* = 0.9961; nGD vs. hCD, *P* = 0.1337, Figure 1C).

**Table 2:** The association between miR-4443 and classic GD clinical parameters by Spearman correlation and multiple stepwise linear regression analysis.

GD parameters	*r*	*P*	β ± SE	*P*
FT3	0.350	0.025		
FT4	0.328	0.036		
sTSH	−0.022	0.889		
TRAb	0.338	0.031	0.042 ± 0.014	0.005
TPOAb	0.047	0.793		
TGAb	−0.10	0.953		

*r, correlation coefficient; β, regression coefficient*.

We then evaluated whether miR-4443 upregulation in uGD patients is a consequence of hyperthyroidism or T cell hyperactivity. We isolated fresh CD4+ T cells from healthy individuals and cultured them with or without T3 treatment. After 24 h or 7 days of cell culture, no significant change was found. Activation of CD4+ T cells by anti-CD3/CD28 antibodies did not influence miR-4443 levels either (Figure S1 in Supplementary Material).

### miR-4443 As a Positive Regulator of CD4+ T Cell Proliferation and Function

**Figure 2:**
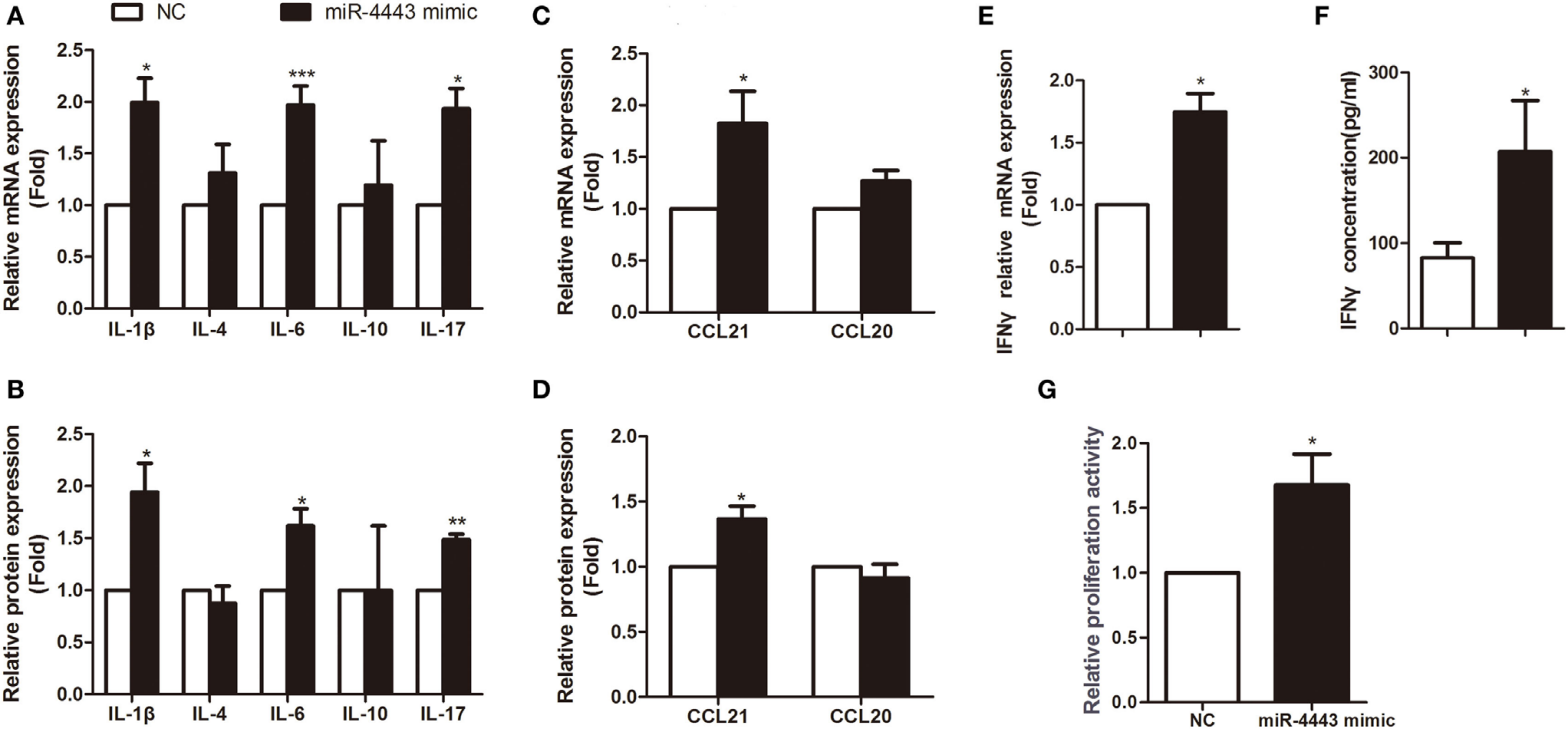
Overexpression of miR-4443 in normal CD4+ T cells increased T cell function and proliferation. CD4+ T cells isolated from healthy donors were transfected with negative controls or miR-4443 mimics. Twelve hours after transfection, the cells were stimulated with anti-CD3 and anti-CD28 antibodies. **(A,C)**. Relative mRNA expression levels of interleukins (IL-1β, IL-4, IL-6, IL-10, IL-17) and chemokines (CCL21, CCL20) in CD4+ T cells. **(B,D)** Relative protein levels of interleukins (IL-1β, IL-4, IL-6, IL-10, IL-17) and chemokines (CCL21, CCL20) in the medium of cultured CD4+ T cells. **(E,F)** Relative mRNA expression and protein levels of IFN-γ. **(G)** Relative proliferation activity of cultured CD4+ T cells. Bars show the mean ± SD of three independent experiments. *P < 0.05; **P < 0.01; ***P < 0.001.

**Figure 3:**
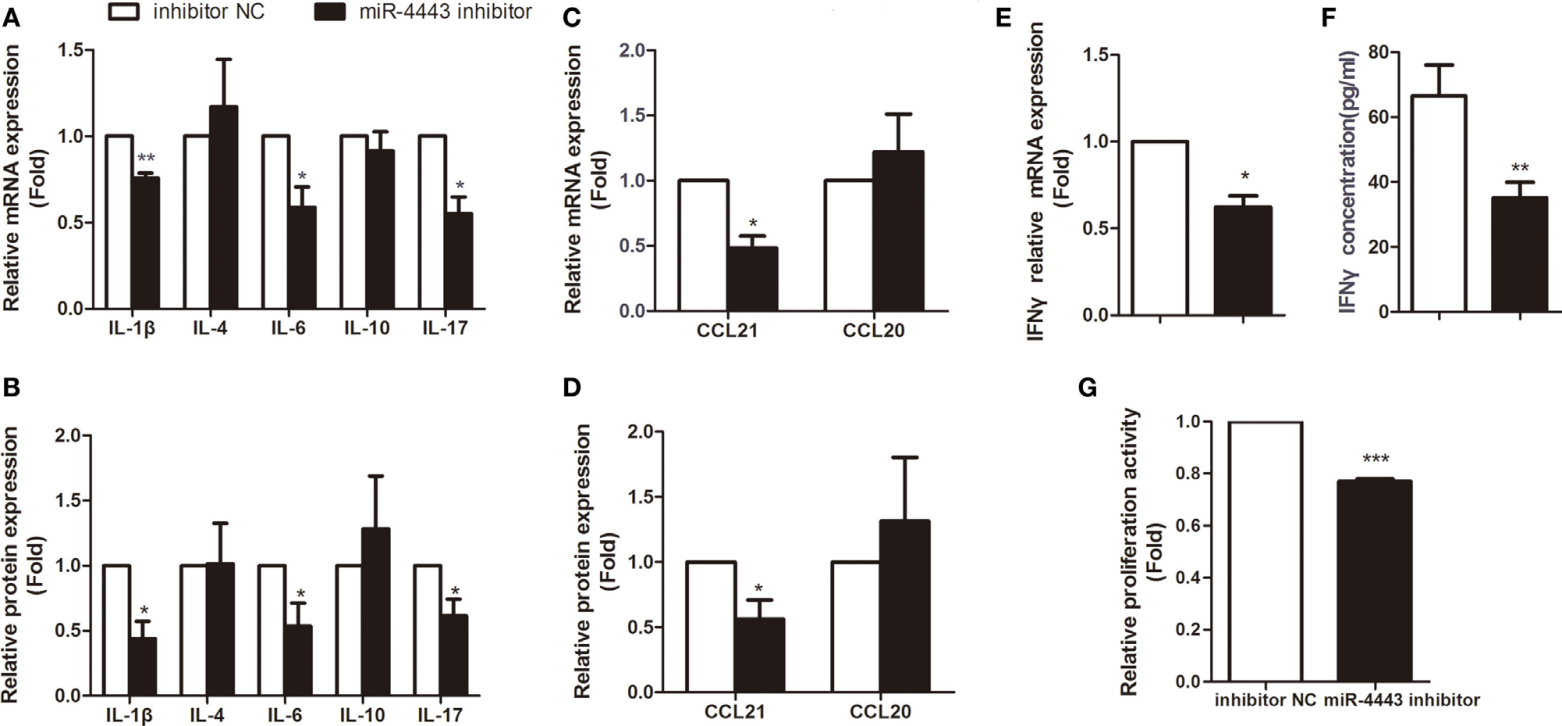
Inhibiting miR-4443 expression in CD4+ T cells isolated from untreated Graves’ disease (uGD) patients downregulated T cell function and proliferation. CD4+ T cells isolated from GD patients were transfected with inhibitor negative controls or miR-4443inhibitors. Twelve hours after transfection, cells were stimulated with anti-CD3 and anti-CD28 antibodies. **(A,C)** Relative mRNA expression levels of interleukins (IL-1β, IL-4, IL-6, IL-10, IL-17) and chemokines (CCL21, CCL20) in CD4+ T cells. **(B,D)** Relative protein levels of interleukins (IL-1β, IL-4, IL-6, IL-10, IL-17) and chemokines (CCL21, CCL20) in the medium of cultured CD4+ T cells. **(E,F)** Relative mRNA expression and protein levels of IFN-γ. **(G)** Relative proliferation activity of cultured CD4+ T cells. Bars show the mean ± SD of three independent experiments. *P < 0.05; **P < 0.01; ***P < 0.001.

As shown in Figures 2A,B, overexpression of miR-4443 greatly increased proinflammatory cytokines, including IL-1β, IL-6 and IL-17, at both mRNA level and protein concentration. The expression of CCL21 was increased compared with that of the negative controls (Figures 2C,D). Both the mRNA and protein levels of IFNγ were upregulated in the miR-4443 mimic group (Figures 2E,F). Meanwhile, we found the plasma concentrations of IL-1β, IL-6, IL-17, CCL21, and IFNγ were higher in uGD than in eGD and nGD (Figure S2 in Supplementary Material). As illustrated in Table S2 in Supplementary Material, IL-6 and IFNγ concentrations correlated significantly with miR-4443 levels, IL-1β, IL-17, and CCL21 levels also have correlation trend with miR-4443 levels while *P* values > 0.05. Moreover, CCK8 assay showed miR-4443 mimics increased CD4+ T cells proliferation (Figure 2G). Consistent with these findings, miR-4443 inhibitors significantly decreased the levels of cytokines, chemokines, and the proliferation of CD4+ T cells obtained from uGD patients (Figure 3).

### Targeting TRAF4 by miR-4443 in CD4+ T Cells

**Figure 4:**
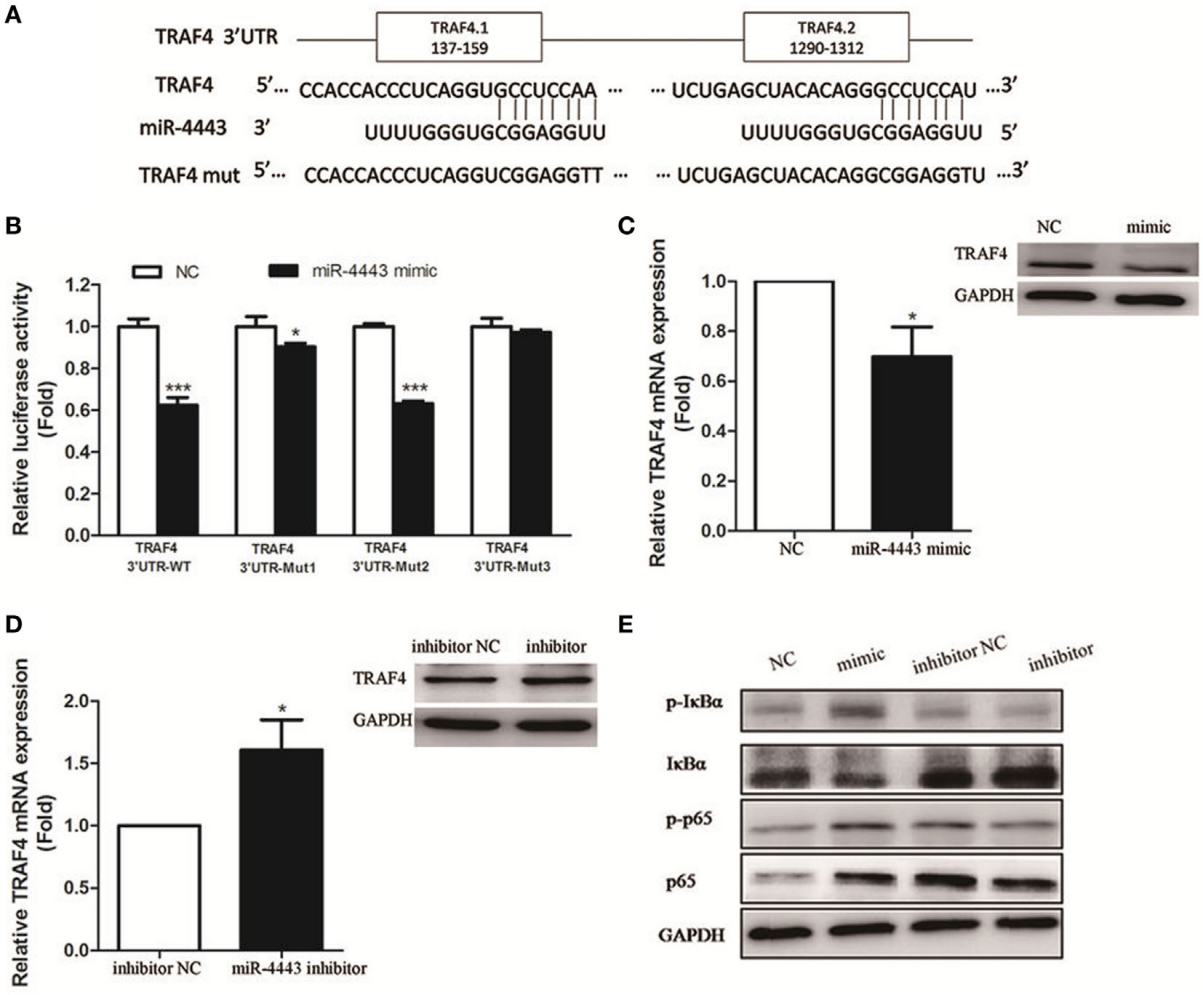
TNFR-associated factor 4 (TRAF4) is a direct target of miR-4443. **(A)** The wild-type or mutant putative target sites for miR-4443 in the 3′UTR of TRAF4 were cloned into downstream of the luciferase gene. **(B)** Luciferase activity was performed by cotransfection with wild-type or mutated TRAF4 3′UTR in the presence of miR-4443 mimics or controls in 293 T cells. Renilla luciferase activities were normalized to firefly luciferase activities and were determined 48 h after transfection. **(C)** TRAF4 mRNA and protein levels in healthy CD4+ T cells after transfection with miR-4443 mimics or controls were detected. **(D)** TRAF4 mRNA and protein levels in untreated Graves’ disease (uGD) CD4+ T cells after transfection with miR-4443 inhibitors or controls were detected. **(E)** Effect of miR-4443 on the activities of NF-κB signaling. Bars show the mean ± SD of three independent experiments. *P < 0.05; ***P < 0.001.

TNFR-associated factor 4, an inhibitor of inflammatory responses, was identified as one of the potential targets of miR-4443 according to TargetScan and miRDB. We constructed plasmids with the TRAF4 3′UTR including the wild-type sequence and mutated sequence (Figure 4A). Luciferase reporter assay showed that the WT luciferase activity, rather than Mut3 luciferase activity, was significantly reduced. miR-4443 binds more at the first putative binding sites because miR-4443 inhibited the luciferase activity of the Mut2 but had little inhibitory effect on the Mut1 luciferase activity (Figure 4B).

In CD4+ T cells transfected with miR-4443, TRAF4 mRNA and protein level was decreased (Figure 4C). In contrast, miR-4443 inhibitors increased the TRAF4 mRNA and protein expression in CD4+ T cells from uGD patients (Figure 4D). Given that TRAF4 is a crucial inhibitor of the NF-κΒ pathway (12–14), we assessed the activity of key players of NF-κΒ pathway. As shown in Figure 4E, miR-4443 mimics increased phosphorylation of p65 (p-p65), total p65, and phosphorylation of IκBα (p-IκBα) levels, while reduced IκBα expression in CD4+ T cells from healthy controls. However, miR-4443 inhibitors downregulated p-p65, p65, and p-IκBα expression, while promoted IκBα expression in CD4+ T cells from uGD patients.

### TRAF4 Is Inversely Correlated with miR-4443 in GD

**Figure 5:**
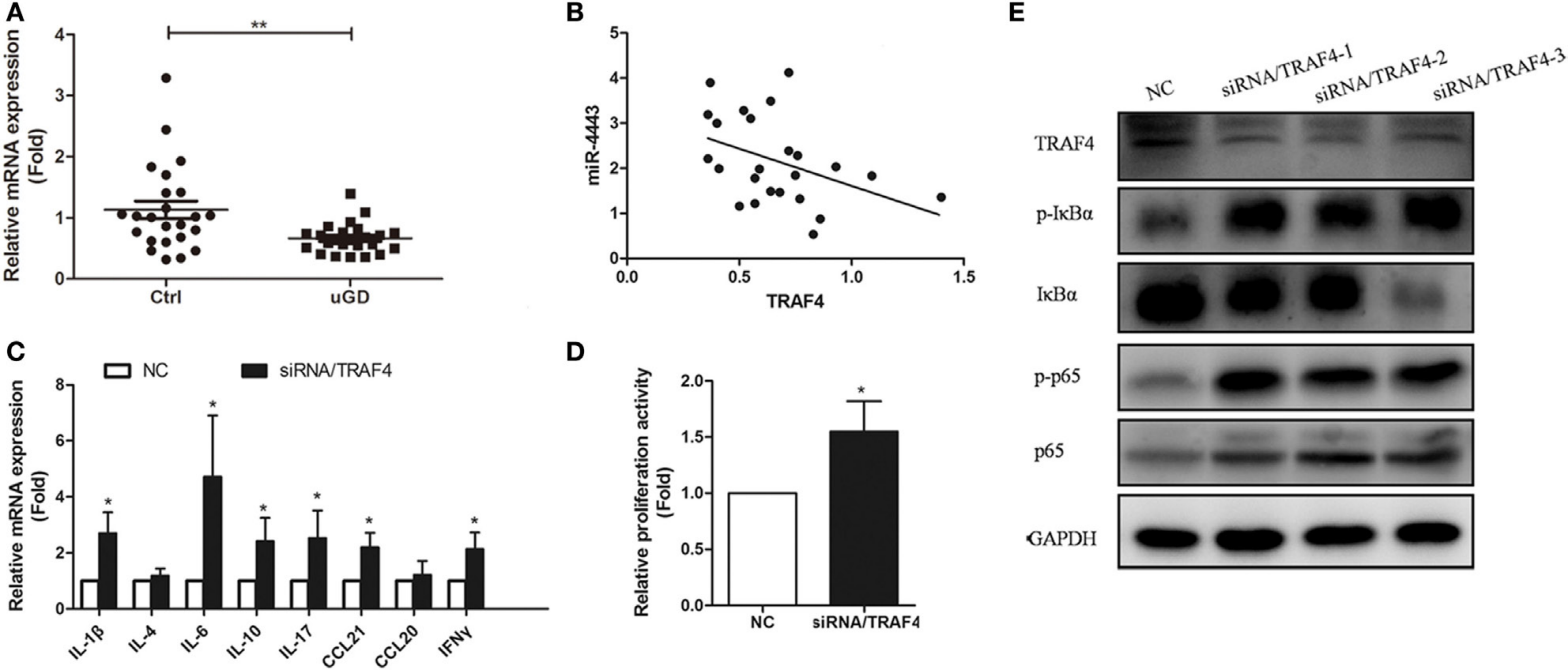
Expression and function of TNFR-associated factor 4 (TRAF4) in Graves’ disease (GD) CD4+ T cells. **(A)** The expressions of TRAF4 in CD4+ T cells from patients with uGD and normal controls were measured by real-time polymerase chain reaction (PCR). **(B)** Correlation between the expression of TRAF4 and the level of miR-4443 in primary CD4+ T cells of patients with GD. **(C,D)** Effect of the TRAF4 knockdown on regulation CD4+ T cell secretion function **(C)** and proliferation **(D)**. **(E)** Effect of TRAF4 knockdown on the activities of NF-κB signaling. Bars show the mean ± SD of three independent experiments. *P < 0.05; **P < 0.01.

As shown in Figure 5A, TRAF4 mRNA levels were significantly downregulated in CD4+ T cells isolated from uGD patients (*P* = 0.037). Moreover, miR-4443 levels were inversely correlated with TRAF4 expression (*r* = −0.432, *P* = 0.035, Figure 5B). Furthermore, knock-down of TRAF4 expression had an effect similar to that of miR-4443 overexpression (Figures 5C–E), the expression levels of cytokines and chemokines were increased, the proliferation of CD4+ T cells was upregulated, and the NF-κΒ pathway was activated.

## Discussion

In this study, we indicated miR-4443 was elevated in UGD patients and was significantly correlated with GD development. *In vitro* study, it demonstrated that miR-4443 induced the overexpression of cytokines, chemokines and the proliferation of CD4+ T cells, which could be explained by activated NF-κΒ pathway *via* targeting TRAF4.

MicroRNAs have been implicated in the pathogenesis of GD. Our previous study revealed that differentially expressed miRNAs in PBMCs were associated with GD and T3 exposure (15). A study subsequently indicated the expression profiles of mRNA/miRNA in Tregs might play important roles in GD development (16). Bernecker et al. found miR-200a decreased in CD4+ T cells from GD (17). However, the expression profile of miRNAs in CD4+ T cells from uGD patients and the effect of certain miRNA on GD CD4+ T cells have not been reported. In this study, we identified for the first time that miR-4443, miR-10, and miR-125b were differentially expressed between uGD patients and healthy controls. Further study confirmed miR-4443 was strongly correlated with clinical GD parameters including FT3, FT4, especially the TRAb levels. After MMI treatment, miR-4443 levels decreased in EGD patients and TRAb-negative GD patients. Unlike previous study, we found there was no significant difference in the expression of miR-200a between uGD patients and controls by both microarray and RT-PCR analysis. This may be due to larger sample size and strict grouping in the current study.

Several miRNAs (e.g., miR-181c, miR-568) levels have already been affected during T cell activation (18, 19). However, the overexpression of miR-4443 that we observed in this study is not likely to be a downstream consequence of increased T lymphocyte activity because miR-4443 levels were not affected by anti-CD3/CD28 antibodies. Besides, elevated concentrations of T3 and T4 are the major cause of GD signs and symptoms, and the effects of T3 on target tissues are roughly four times more potent than those of T4 (20). Interestingly, we previously found T3 treatment could directly affect the expression of miRNAs in cultured PBMCs from healthy subjects (15). However, T3 was not likely to change the miR-4443 levels in this study. Therefore, miR-4443 may be a potential cause of GD.

MicroRNAs are important regulators of T cell activation, proliferation and the cytokine production. For example, miR-125a, miR-21, miR-31, miR-23b, and miR-142-3p/5p can alter the expression of proinflammatory and anti-inflammatory cytokines *via* immune pathways (9, 21–23). It was previously found that miR-4443 expressed differently in EV71-infected human rhabdomyosarcoma (RD) cells and uninfected RD cells (24). Shefler et al. recently reported that miR-4443 presented in microvesicles derived from activated T cells could regulate mast cell activation by targeting PTPRJ gene (25). These suggested that elevated miR-4443 in GD CD4+ T cells plays a potential role in inflammation response. Importantly, we observed that overexpression of miR-4443 in normal CD4+ T cells increased GD-related cytokines including IL-1β, IL-6, IL-17, CCL21, and IFN-γ production. In contrast, inhibition of miR-4443 in uGD CD4+ T cells reduced these cytokines expression. IL-1β is a proinflammatory cytokine, which has variety of effects on GD, such as modification of thyroid epithelial tightness and induction of cytokine expression in thyroid cells (26). IFN-γ is important in initiating adaptive immune response, and can stimulate the production of CXC chemokines which are powerful chemotactic factor for recruiting Th1 cells to thyroid gland (3). IL-6, secreted by Th2 cell under humoral response, is involved in the activation of T cells, the production of antibodies by B cells and recruitment of dendritic cells in the thyroid (27, 28). Besides, the percentage of Th17 cell and IL-17 level are associated with the pathogenesis of GD (5). Increasing chemokines are confirmed having crucial roles in GD process. Our previous study showed that CCL21 was increased in UGD patients. It may consequently induce circulation of CC-chemokine receptor 7 (CCR7)-expressing cells to thyroid gland (29). It is likely that these increased expression cytokines are released into the circulatory system and up taken by thyroid gland to participate in the local immune response. Meanwhile, the percentage of CD4+ T cells and the ratio of CD4+/CD8+ cells were higher in both GD and Graves’ ophthalmopathy than those of healthy controls (30). Our present study found overexpression of miR-4443 could promote CD4+ T cell proliferation and inhibition of miR-4443 level could reduce proliferation. Taken together, miR-4443 participated in the GD pathogenesis through increasing cytokines secretion and promoting CD4+ T cell proliferation.

MicroRNAs bind to “seed” sequences in target mRNAs, leading to reduced expression of target mRNAs. According to bioinformatics tools, TRAF4 which could attenuate inflammatory responses was identified as a potential target of miR-4443. Moreover, there are two putative binding sites in the 3′UTR of TRAF4. With the dual-luciferase reporter assay, we indicated that miR-4443 mainly targeted the first binding site (positions 152–159 of TRAF4 3′UTR). Moreover, upregulation of miR-4443 reduced the expression of TRAF4 in healthy CD4+ T cells. The opposite effect was observed when downregulate miR-4443 level in uGD CD4+ T cells. We further found the expression of TRAF4 was decreased in uGD and was negatively correlated with miR-4443 level. All these results suggested that TRAF4 was a direct target gene of miR-4443 in GD CD4+ T cell. Interestingly, one recent study also found that miR-4443 acts in a tumor-suppressive manner by down-regulating TRAF4, which further confirms our findings (31).

TNFR-associated factor 4 is an atypical member of the TRAF superfamily (32). Unlike the other members, TRAF4 negatively regulates immune signaling. TRAF4 interacts with NOD2, TRAF6 and TRIF to inhibit NF-κΒ activation (12–14). NF-κΒ signal pathway is a major regulator of innate and adaptive immune responses (33). The classical NF-κΒ pathway is induced by signals including antigens, TLR ligands and cytokines, involved in controlling inflammation, survival and proliferation (34). Ligands for TNF receptor superfamily members engage the alternate NF-κΒ pathway which is crucial for lymphoid organogenesis. Marinis et al. found IKKα’s phosphorylation of serine-426 on TRAF4 was required for the negative regulation of TRAF4 and also found macrophages stably expressing TRAF4 had dampened NF-κΒ responses, indicated by decreased IκBα phosphorylation (35). In experimental autoimmune encephalomyelitis model, the TRAF4-deficient mice had increased numbers of immune cell infiltration in the brain and a significantly higher expression of proinflammatory genes compared with that in the control mice (36). Similar result was observed in the keratinocyte of TRAF4-deficient mice, showing basal-level and IL-17–induced activation of ERK1/2, JNK, P65, and p38 increased (37). Our results showed that the change of miR-4443 levels affected the NF-κΒ pathway activity. We subsequently observed TRAF4 function in CD4+ T cells, finding cytokines production and CD4+ T cell proliferation was increased obviously when silence the TRAF4 expression in CD4+ T cell by NF-κΒ pathway. Thus, we concluded that miR-4443 binds TRAF4 to activate NF-κΒ signal pathway, leading to aberrant T cell function.

One major limitation of this study is the relatively small sample size. Further long-term prospective investigations with larger sample size are needed to identify miR-4443 as biomarkers. Besides, we did not demonstrate that miR-4443 seems specific to GD because we did not investigate the miR4443 expression in CD4+ T cells from patients with other autoimmune disease.

In conclusion, we first revealed that miR-4443 levels were increased in CD4+ T cells from uGD patients and strongly associated with clinical parameters of GD. We also found *in vitro* assays that ectopic expression of miR-4443 targeted TRAF4 to induce aberrant CD4+ T cells cytokines secretion and proliferation through NF-κΒ pathway. These findings revealed that elevated miR-4443 contributes to the immune-pathogenesis of GD.

## Author Contributions

Conceptualization: SW and WW. Data curation: YQ and YZ. Formal analysis: YQ and FH. Funding acquisition: SW. Investigation: YQ and XC. Methodology: XC and QZ. Project administration: SW, WW, and GN. Resources: LY and DL. Software: BC. Supervision: SW. Validation: YQ and YZ.

## Conflict of Interest Statement

The authors declare that the research was conducted in the absence of any commercial or financial relationships that could be construed as a potential conflict of interest.

## Supplementary Material

Figure S1 **(A)** Expression of miR-4443 in CD4+ T cells from healthy human with or without T3 treatment. **(B)** Expression of miR-4443 in CD3/CD28 stimulated CD4+ T cells from healthy human. Bars show the mean ± SD of three independent experiments.

Figure S2 Plasma concentrations of IL-1β, IL-6, IL-17, CCL21, and IFNγ. Bars show the mean ± SD. ***P* < 0.01; ****P* < 0.001.
