# Supplementary figures and images for: MicroRNA-4443 Causes CD4+ T Cells Dysfunction by Targeting TNFR-Associated Factor 4 in Graves’ Disease
Source: Front Immunol. 2017 Nov 1;8:1440. doi: 10.3389/fimmu.2017.01440 (PMC5671953; doi:10.3389/fimmu.2017.01440)

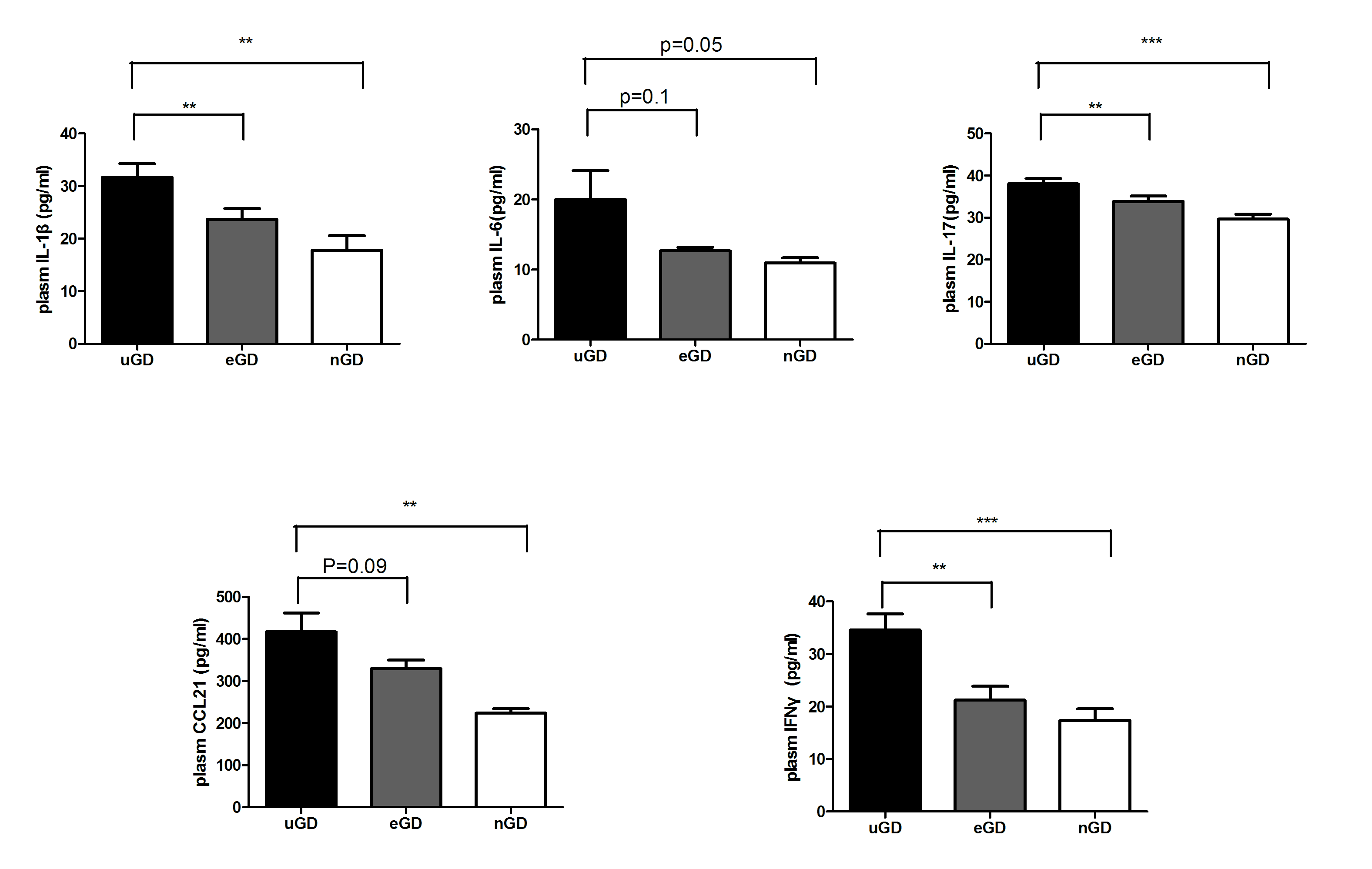

Supplement: Figure S2 — Plasma concentrations of IL-1β, IL-6, IL-17, CCL21, and IFNγ. Bars show the mean ± SD. **P < 0.01; ***P < 0.001. [file image_2.tif]
