# Supplementary material for: MicroRNA-4443 Causes CD4+ T Cells Dysfunction by Targeting TNFR-Associated Factor 4 in Graves’ Disease
Source: Front Immunol. 2017 Nov 1;8:1440. doi: 10.3389/fimmu.2017.01440 (PMC5671953; doi:10.3389/fimmu.2017.01440)
Supplement: Supplementary file 3 [file table_1.docx]

**Table S1.** Primer sequences used for quantitative PCR

| Gene Symbol | Primer sequence |
| --- | --- |
| β-actin | F: 5'-CATGTACGTTGCTATCCAGGC-3' |
|  | R: 5'-CTCCTTAATGTCACGCACGAT-3' |
| IL-1β | F: 5'-ATGATGGCTTATTACAGTGGCAA-3' |
|  | R: 5'-GTCGGAGATTCGTAGCTGGA -3' |
| IL-4 | F: 5'- ATGGGTCTCACCTCCCAACT-3' |
|  | R: 5'-GATGTCTGTTACGGTCAACTCG-3' |
| IL-6 | F: 5'-ACTCACCTCTTCAGAACGAATTG-3' |
|  | R: 5'-CCATCTTTGGAAGGTTCAGGTTG-3' |
| IL-10 | F: 5'-TCAAGGCGCATGTGAACTCC-3' |
|  | R: 5'-GATGTCAAACTCACTCATGGCT-3' |
| IL-17A | F: 5'- AGATTACTACAACCGATCCACCT-3' |
|  | R: 5'-GGGGACAGAGTTCATGTGGTA-3' |
| CCL21 | F: 5'- GTTGCCTCAAGTACAGCCAAA-3' |
|  | R:5'- AGAACAGGATAGCTGGGATGG-3' |
| CCL20 | F:5'- TGCTGTACCAAGAGTTTGCTC-3'  R:5’-CGCACACAGACAACTTTTTCTTT-3’ |
| IFN-γ | F: 5’-TCGGTAACTGACTTGAATGTCCA-3'  R:5'-TCGCTTCCCTGTTTTAGCTGC-3' |
