# Supplementary material for: MicroRNA-4443 Causes CD4+ T Cells Dysfunction by Targeting TNFR-Associated Factor 4 in Graves’ Disease
Source: Front Immunol. 2017 Nov 1;8:1440. doi: 10.3389/fimmu.2017.01440 (PMC5671953; doi:10.3389/fimmu.2017.01440)
Supplement: Supplementary file 4 [file table_2.docx]

**Table S2.**Correlation between miR-4443 level and expressions of cytokines

| Cytokines | *r* | *p* |
| --- | --- | --- |
| IL-1β | 0.516 | 0.059 |
| IL-6 | 0.81 | <0.01 |
| IL-17 | 0.435 | 0.12 |
| CCL21 | 0.416 | 0.14 |
| IFNγ | 0.548 | 0.042 |
